# Uncovering the Power of GPR18 Signalling: How RvD2 and Other Ligands Could Have the Potential to Modulate and Resolve Inflammation in Various Health Disorders

**DOI:** 10.3390/molecules29061258

**Published:** 2024-03-12

**Authors:** Ewelina Honkisz-Orzechowska, Dorota Łażewska, Grzegorz Baran, Katarzyna Kieć-Kononowicz

**Affiliations:** Department of Technology and Biotechnology of Drugs, Faculty of Pharmacy, Jagiellonian University Medical College, 9 Medyczna St., 30-688 Krakow, Poland; dorota.lazewska@uj.edu.pl (D.Ł.); gbaran.baran@student.uj.edu.pl (G.B.)

**Keywords:** GPR18, agonist, antagonist, resolvin D2, inflammation, resolution

## Abstract

The resolution of inflammation is the primary domain of specialised pro-resolving mediators (SPMs), which include resolvins, protectins, and their forms synthesised under the influence of aspirin and the maresins. The role of these SPMs has been discussed by many authors in the literature, with particular reference to neuroinflammation and significant neurological disorders. This review discusses the role of G protein-coupled receptor 18 (GPR18), resolvin D2 (RvD2) activity, and the GPR18-RvD2 signalling axis, as well as the role of small molecule ligands of GPR18 in inflammation in various health disorders (brain injuries, neuropathic pain, neurodegenerative/cardiometabolic/cardiovascular/gastrointestinal diseases, peritonitis, periodontitis, asthma and lung inflammation, Duchenne muscular dystrophy, SARS-CoV-2-induced inflammation, and placenta disorders. The idea of biological intervention through modulating GPR18 signalling is attracting growing attention because of its great therapeutic potential. With this paper, we aimed to present a comprehensive review of the most recent literature, perform a constructive view of data, and point out research gaps.

## 1. Introduction

### 1.1. Inflammation Sequence

Inflammation is the body’s natural protection process that eliminates pathogens and maintains homeostasis. This natural and protective action occurs in response to specific factors such as injury (cuts, surgical wounds, overuse), infections (bacterial, viral, and fungal), or a pro-inflammatory diet. The normal ageing process is also reported in this group of factors, as it is associated with a greater vulnerability of tissues and organs to oxidative stress and redox disturbances [1]. When inflammation is not resolved correctly, it can lead to tissue damage and pain. Furthermore, it might turn into chronic inflammation with progression to chronic diseases and is associated with complications related to wound healing, cardiometabolic diseases (cardiovascular disease and diabetes), mild cognitive decline and mood disorders, pulmonary diseases (allergy, asthma), and arthritis. The critical point is that the appearance of inflammation is only a general statement. The dynamic and severity of inflammation can give a picture of the scale of its destructive nature. Prolonged inflammation makes it challenging for the immunological circuit to develop the mechanism that results in restitutio ad integrum.

Several stages comprise the resolution process. First, the resolution of oedema (the medical term for swelling) is the vascular response to inflammatory stimuli. The release of molecules associated with oedema is triggered, and mast cell degranulation is a crucial source of histamine that leads to vasodilation and increased permeability of blood vessels [2]. Macrophages are also a primary source of pro-inflammatory interleukin-1 (IL-1) [3]. Enzyme degradation and chemical instability lead to a limited lifespan for the following secreted molecules: histamine 2 h, IL-1 2.5 h. Over time, the molecules break down, thereby creating a self-limiting process. Inflammatory signalling also triggers a counteracting anti-inflammatory response through glucocorticoids and the pituitary-adrenal axis [4]. Second, the neutrophil resolution, categorised as the initial response, can be harmful. Under the influence of inflammatory mediators, adhesion molecules appear on the surface of the endothelium. Neutrophils adhere to the vasculature adhesion molecules and finally migrate to sites of inflammation. When neutrophils degranulate, they produce myeloperoxidase (MPO) and neutrophil elastase, which help deactivate the inflammatory signalling molecules and damage-associated molecular patterns (DAMPs) [5]. Third, if, for some reason, the macrophage resolution does not work, it causes chronic inflammation. Macrophages are master controllers of innate immune cells. Because these cells are long-lived inflammatory cells, their phenotype should be changed to an anti-inflammatory phenotype for which IL-10 secretion is characteristic. Furthermore, in the resolution phase, macrophages also play a role in returning the tissue to its normal state [3].

### 1.2. Modulators of the Inflammatory Response

To avoid harmful effects, inflammation should be limited in time. In this context, the biosynthesis of active mediators promotes the return to homeostasis by acting on critical inflammation events [6]. The process of returning to homeostasis is called the resolution of inflammation. It is important to emphasise that it is not a passive process in the sense of a self-dissolving process. Recent evidence has shown that inflammation resolution has unique mechanisms of response and action, different from the causes and onset of the inflammatory response. Many anti-inflammatory therapies target the inhibition of mediators, such as cyclooxygenases or TNF-α, or the antagonism of receptors, such as glucocorticosteroids. However, specialised pro-resolving mediators (SPMs) focus on supporting the body’s natural processes to clear inflammatory components and resolve the inflammatory process [7]. The discovery of these genus SPMs has provided proof that the resolution phase is orchestrated by local mediators and their biosynthesis from n-3 precursors eicosapentaenoic acid (EPA) and docosahexaenoic acid (DHA) [8]. The role of SPMs, including resolvins, protectins, and their forms that are synthesised after aspirin intervention, as well as the maresins, has been discussed by a significant number of authors in the literature, with particular reference to neuroinflammation and central neurological disorders (see review [9]). It is interesting to note that multiple SPMs frequently activate the same receptor and that a single SPM can work by activating multiple receptors [10].

### 1.3. G-Protein Coupled Receptor 18 (GPR18)

**Figure 1:**
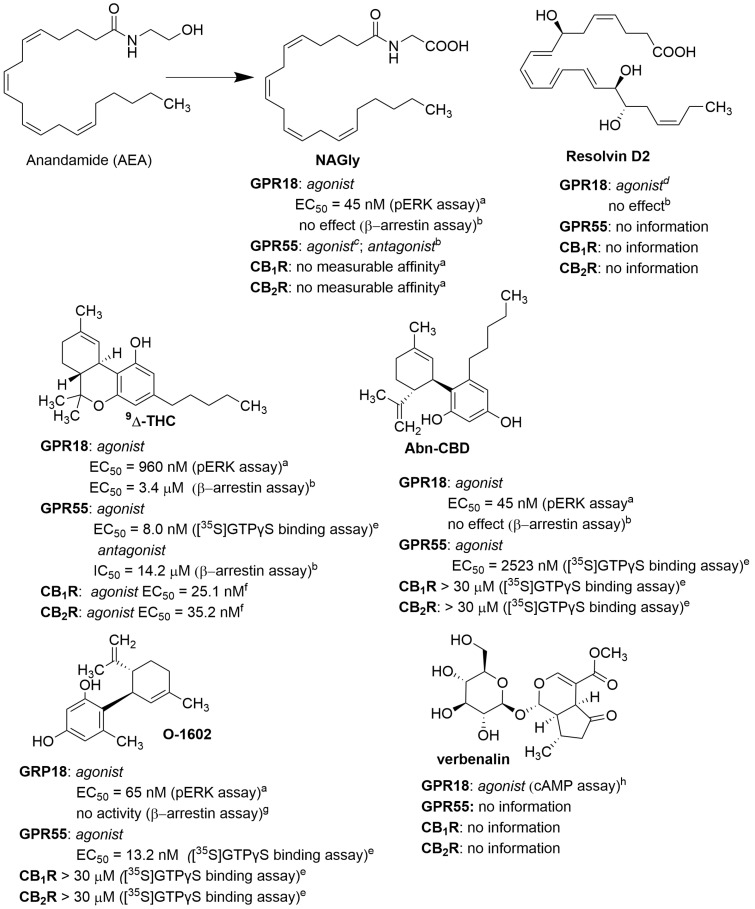
Structures of endogenous and natural agonists of GPR18. Data from ^a^ [18], ^b^ [19], ^c^ [20], ^d^ [16], ^e^ [21], ^f^ [22], ^g^ [13], ^h^ [23].

GPR18 has a structure of 331 amino acids that form seven transmembrane domains, is encoded on chromosome 13, and its transcripts have been found in a variety of human and rodent lymphoid tissues, including the spleen, thymus, peripheral blood leukocytes, small intestine, appendix, and lymph node [11,12]. Immunohistochemical staining analysis revealed the presence of the GPR18 receptor in both the cell membrane and inside the cells [13]. Since GPR18 plays a significant role in the resolution stage of an inflammatory response [14], resolvin D2 (**RvD2**, Figure 1) deserves special attention as an SPM [15]. We decided to focus on the **RvD2** because it has been previously reported that it selectively activates GPR18 in a β-arrestin–based ligand-receptor interaction system [16]. In the cited study, it was demonstrated that Chinese hamster ovary cells (CHO) overexpressing human GPR18 demonstrated distinct chemiluminescence signal responses after treatment with **RvD2** (10^−13^–10^−8^ M) in a dose-dependent manner with EC_50_ values of 2.0 × 10^−13^ M. Although some researchers did not observe the effect of GPR18 stimulation by **RvD2** in their work, many functional studies using animal models implicate **RvD2** in processes that resolve inflammation [17]. 

## 2. GPR18 Ligands

Not many natural or synthetic compounds have been described as ligands for GPR18 so far. In 2020, Morales et al. discussed the structures of such compounds in an interesting review [24]. Our paper focuses on new ligands and describes some previously known compounds in more detail. The described ligands were divided into agonists and antagonists, even though complex pharmacology was apparent due to constitutive activity or different effects depending on the assays used. Some ligands showed a stimulatory effect on a specific signalling cascade (e.g., via β-arrestin or Gα and Gβγ units) but not on both pathways simultaneously. This was the case for *N*-arachidonoylglycine (**NAGly**; Figure 1), where an agonist effect was observed in the calcium activation assay or the pERK assay but not in the β-arrestin assay. For some synthetic ligands, different pharmacological effects were observed in different assays, such as agonist-like behaviour in one pathway and inverse agonist-like behaviour in another. In principle, the only compound for which (based on published data to date) the same pharmacological effect has been observed (irrespective of the type of assays) is Δ^9^-tetrahydrocannabinol (**Δ^9^-THC**; Figure 1).

### 2.1. GPR18 Agonists

Stimulation of GPR18 is induced by endogenous ligands such as lipid acid derivatives (**NAGly**, **RvD2**) or cannabinoids (**Δ^9^-THC** or abnormal cannabidiol (Abn-CBD)). These ligands are generally non-selective and have pharmacological effects on other biological targets, such as cannabinoid receptors (CB_1_, CB_2_) or GPR55. Kohno et al. described **NAGly** as the endogenous GPR18 agonist in 2006. This ligand is formed in the body by metabolic conversion of the known cannabinoid agonist, anandamide (**AEA**; Figure 1), and lacks affinity for CB_1_ and CB_2_ [25].

**Figure 2:**
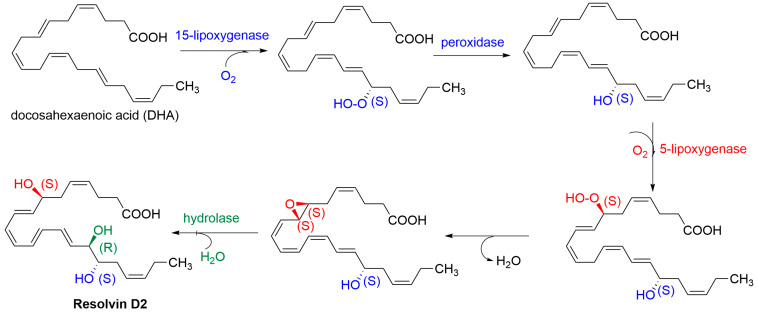
Biosynthesis of RvD2.

In 2015, Chiang et al. showed that **RvD2** is an agonist of GPR18 (Figure 1) [16]. **RvD2** is biosynthesised from DHA by enzymatic reactions involving, in particular, lipoxygenases (15 and 5), yielding a trihydroxy derivative (7S, 16R, 17S) in the final hydrolysis step (Figure 2) [26].

**Figure 3:**
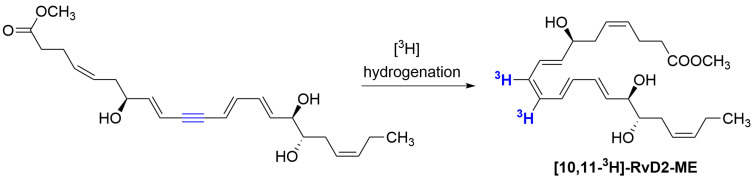
Synthesis of radiolabelled derivative of **RvD2** ([10,11-^3^H]-RvD2-ME).

To confirm that **RvD2** binds directly to GPR18, Chiang et al. synthesised tritiated **RvD2** at positions 10 and 11, as shown in Figure 3. The obtained radioligand, [10,11-^3^H]-**RvD2-ME** (Figure 3), was used to bind the recombinant human GPR18 expressed in CHO cells. Specific binding was achieved with a K_d_ value of 9.6 ± 0.9 nM [16].

**Figure 4:**
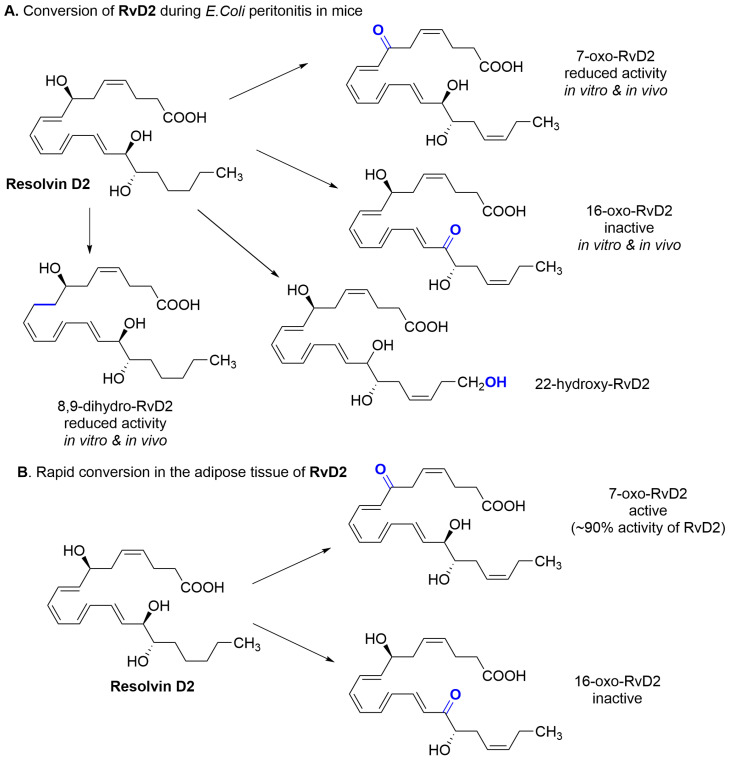
Observed metabolism of RvD2.

**NAGly** and **RvD2** are ligands whose agonistic effect on GPR18 has been questioned by some researchers, as they did not observe this agonistic effect in their studies [27]. The reason for this may be, especially in the case of **RvD2**, the low metabolic stability of this ligand. **RvD2** is a polyunsaturated DHA acid derivative formed in the body by a series of enzymatic reactions. In rodent and isolated human cell studies, **RvD2**, like other resolvins, is inactivated by local metabolic pathways. Serhan’s group showed the formation of **RvD2** metabolites that exhibited weaker or no pharmacological activity [28]. In a study in the mice model of *E. coli*-induced peritonitis, the formation of 7-oxo-RvD2, 16-oxo-RvD2, 22-hydroxy-RvD2, and 8,9-dihydro-RvD2 derivatives was observed (Figure 4A). The 7-oxo-RvD2 and 8,9-dihydro-RvD2 derivatives showed weaker pharmacological effects than RvD2 (in vivo—anti-inflammatory; in vitro—inhibition of human neutrophil chemotaxis and macrophage phagocytosis), whereas 16-oxo-RvD2 was generally inactive.

Additionally, the formation of 10E isomer was observed, which caused an essential reduction of **RvD2** activity [26]. It indicates that the appropriate geometry of endogenous **RvD2** is essential for bioactivity. In other studies conducted in obese mice, the **RvD1** and **RvD2** reduced inflammation in obese adipose tissues but were quickly dehydrogenated (Figure 4B) [29]. In the case of RvD2, two metabolites were observed: 7-oxo-RvD2 and 16-oxo-RvD2. The 7-oxoRvD2 product retained approximately 90% of **RvD2** activity but may undergo subsequent further metabolism, leading to its complete inactivation.

**Δ^9^-THC** (Figure 1) is one of the agonists most commonly used in studies related to GPR18, although this ligand is not selective as it also shows activity at the cannabinoid receptors (CB_1_ and CB_2_) and GPR55. Other commonly used GPR18 agonists are synthetic cannabidiol derivatives such as **Abn-CBD** or **O-1602** (Figure 1). They are not active at cannabinoid receptors CB_1_ and CB_2_ but show activity at GPR55. Whereas **Abn-CBD** is a relatively weak activator for both receptors (GPR18: EC_50_ < 835 nM; GPR55: EC_50_ = 2523 nM), **O-1602** shows activity for them in the low nanomolar range (GPR18: EC_50_ = 65 nM; GPR55: EC_50_ = 13 nM) [30].

Recently, studies conducted by Yuan et al. revealed that verbenalin (Figure 1), an iridoid glucoside, is a GPR18 agonist [23]. Verbenalin is present in *Verbena* (*Verbena officinalis* L.), a popular herb that grows in Europe, Asia, and Africa, although it comes from America. *Verbena* has been known to show anti-inflammatory activity for many years [31]. Yuan et al. confirmed the GPR18 agonist activity of verbenalin in the cAMP test and believe that part of its anti-inflammatory effect is related to the activation of GPR18. 

**Figure 5:**
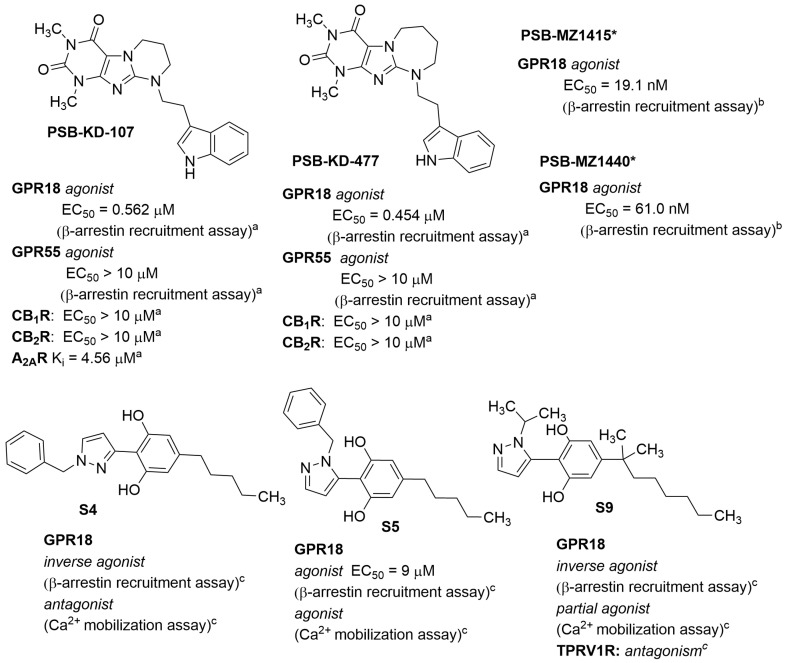
Structures of synthetic GPR18 agonists. * The structures are undisclosed and will be published elsewhere. Data from: ^a^ [19]; ^b^ [32];^c^ EP3901142.

The structures of the first synthetic non-lipidic GPR18 agonists were described by Schoeder et al. [19]. These compounds are tricyclic xanthine derivatives with greater agonist potency than **Δ^9^-THC** (β-arrestin recruitment assay) and selectivity towards CB_1_, CB_2_, and GPR55 receptors. Their structures are presented in Figure 5. Both **PSB-KD-107** and **PSB-KD-477** showed submicromolar activity for GPR18. Other compounds with agonist activity synthesised by this group are **PSB-MZ1415** (or named **PSB-KK-1415**; structure unpublished; Figure 5) and **PSB-MZ1440** (or named **PSB-KK-1440**; Figure 5). These compounds showed very high activity in the β-arrestin recruitment assay, with an EC_50_ of 19.1 nM and EC_50_ of 61 nM, respectively. Both ligands do not affect GPR55 [32]. 

In a patent application, Jagerovic et al. claimed to have identified other synthetic non-lipid agonists, derivatives of pyrazolylbenzene-1,3-diols [33]. The representative structures are shown in Figure 5. The activity of these compounds was confirmed in calcium ion mobilisation and β-arrestin recruitment assays. The observed activity among tested compounds depended on the used assay. Compound **S4** (Figure 5) was a GPR18 inverse agonist in the ß-arrestin recruitment assay and an antagonist in the Ca^2+^ mobilisation assay (against NAGly). In contrast, compound **S5** (Figure 5) proved to be an agonist in both assays and compound **S9** (Figure 5) showed inverse agonist activity in the β-arrestin recruitment assay and partial agonist activity in the Ca^2+^ mobilisation assay. Surprisingly, a relatively minor structural change in the way in which a benzylpyrazole moiety is attached at position 3 or 5 to the resorcinol ring results in such changes in pharmacological effect, especially in the calcium ion mobilisation assay, i.e., position 3: compound **S4**—antagonist vs. position 5: compound **S5**—agonist.

### 2.2. GPR18 Antagonists

**Figure 6:**
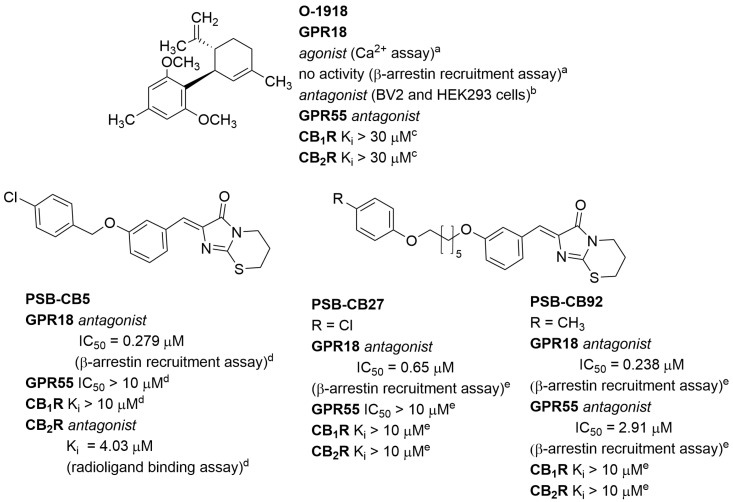
Structures of selected synthetic GPR18 antagonists. Data from: ^a^ [13]; ^b^ [35]; ^c^ [34]; ^d^ [36]; ^e^ [27].

One of the first synthetic ligands described as a GPR18 antagonist was compound **O-1918** (Figure 6) [24]. **O-1918** is commonly used in studies regarding GPR18, although this compound also shows antagonist activity towards the GPR55 receptor (no affinity for CB_1_ and CB_2_ receptors) [34]. Additional studies have shown that this compound behaves as an agonist or antagonist depending on the type of assay applied. **O-1918** acted as an agonist in the calcium mobilisation assay (as did **NAGly**, **Abn-CBD**, **O-1602** and **Δ^9^-THC**) and did not activate signalling through β-arrestin. It is, therefore, a biased ligand [13]. In contrast, in BV-2 and HEK293-GPR18 cells, **O-1918** attenuated or blocked migration induced by **NAGly**, **Abn-CBD** and **O-1602** [35].

The first structures of synthetic selective antagonists were described in 2014 by Rempel et al. [36]. These imidazothiazinone derivatives antagonised **Δ^9^-THC**-induced activity in the β-arrestin recruitment assay, and some of them showed high affinity and good selectivity against CB_1_, CB_2_, and GPR55 receptors. Among them was compound **PSB-CB5** (Figure 6), with an IC_50_ of 279 nM and ≥14-fold selectivity vs. CB_1_, CB_2_, and GPR55. Further structural modifications led to other compounds with high affinity and selectivity, such as **PSB-CB27** and **PSB-CB92** (Figure 6) [27]. Compounds **PSB-CB5** (as **CID-85469571**) and **PSB-CB27** are commercially available as reference antagonists for GPR18 studies.

## 3. RvD2/GPR18 Axis in the Resolution Phase of Inflammation

### 3.1. Brain Injuries

Inflammation in the central nervous system (CNS) is always linked to a disease or disorder. When the brain is inflamed (the term neuroinflammation is commonly used to describe this state), the brain’s immune system becomes overactive. Specialised immune cells (microglia) respond to infection or injury in a healthy brain. For many years, it was believed these cells only became functional when encountering infections or injuries. However, we now know that microglia are active in health and disease [37]. 

The role of microglia in maintaining normal function of the brain is critical, as microglia-mediated neuroinflammation is a shared hallmark of neurodegenerative disorders such as Alzheimer’s disease, Parkinson’s disease, and multiple sclerosis. There are many mechanisms underlying these diseases, and the exact pathological one is unclear, but in general, proteins that do not fold correctly begin to accumulate in the brain and trigger the sequences of unwanted events. Although microglia cells only make up about 5–10% of the total cell population in the brain, they are distributed in different anatomical locations across the CNS, interact with neurons and other glial cells (e.g., in the cerebral cortex), which is crucial for memory and learning (e.g., in the hippocampus), and is involved in motor skills (e.g., in the cerebellum) [38,39]. Microglia are essential for brain health as they continuously remove plaques, damaged neurons, and infectious agents from the CNS. The report highlights the role of GPR18 in microglia neurotoxicity after HIV-1 Tat [40]. In this study of microglia cells, the fatty acid amide hydrolase (FAAH) inhibitor (PF3845) had a neuroprotective effect when incubated with Tat protein, and this effect was blocked by the selective GPR18 antagonist (**PSB-CB5**: **CID-85469571**). These findings suggested that GPR18 has a vital role in regulating activated microglia. RvD2 was also found to play a protective role in early brain injury in an experimental model of subarachnoid haemorrhage (SAH) in rats [41]. In an extensive study, Zhang et al. confirmed the expression of GPR18 in the meninges, hypothalamus, cortex, and white matter but very minor expression in the hippocampus and thalamus. They found that when a rat develops SAH, the expression of GPR18 increases in the meninges and hypothalamus and decreases in the cortex and white matter. In the SAH group, treatment with **RvD2** (0.9 μg/kg) (1) improved neurological evaluation that included six individual tests for motor and sensory functions; (2) attenuated the degranulation of mast cells, which reflected decreased capacity to release a multitude of pro-inflammatory mediators; (3) decreased the expression of matrix metalloproteinase-9 (MMP-9), aquaporin-4 (AQP4)—proteins related to the integrity of blood-brain barrier (BBB), increased expression of which indicates disability of the BBB; (4) reduced the level of cleaved caspase-3 and reactive oxygen species modulator 1 (ROMO1), indicating a protective role against apoptosis and oxidative stress in the cortex, respectively; and (5) decreased the beta-amyloid precursor protein (APP) and increased myelin basic protein (MBP) in the white matter, resulting in reduced axonal and myelin injury associated with SAH [41]. Inflammation also impacts patients with diabetes mellitus (DM) who suffer from acute ischemic stroke (AIS). It was found that macrophages from patients with DM and AIS showed increased inflammation, but **RvD2** treatment reduced this pro-inflammatory response by increasing the CD206/iNOS ratio and down-regulating markers of the mitogen-activated protein kinase (MAPK) and nuclear factor kappa B (NF-κB) pathways. In turn, in a mouse model of DM-related AIS, 1 nM **RvD2** treatment mitigated brain injury, neurological dysfunction, and the inflammatory response [42]. Plasma levels of **RvD2** and one of the classic pro-inflammatory mediators, leukotriene B_4_ (LTB_4_), might be used to indicate post-stroke inflammation. The balance between pro-resolving and pro-inflammatory signals in ischemic stroke was adversely affected by DM, as shown by the significantly decreased ratio in DM stroke patients compared to non-DM stroke diabetic patients [43]. A significant decrease in endogenous RvD2 was also observed in cerebral ischemia/reperfusion (CI/R) injury in Sprague-Dawley rats that were subjected to middle cerebral artery occlusion and reperfusion (MCAO/R) [44]. However, when exogenous **RvD2** was administered at doses of 50 μg/kg and 100 μg/kg, the level of interleukin-6 (IL-6) and tumour necrosis factor-alpha (TNF-α) was significantly lower in infarcted brain tissue following MCAO/R, suggesting a neuroprotective role for **RvD2**.

### 3.2. Neuropathic Pain

Neuropathic pain is a very complex phenomenon with no common origin or location. It is caused by pathological peripheral and central nervous system changes and accompanies many neurological disorders. Neuroinflammation and cytokine signalling are inherent in neuropathic pain [45]. Attention is focused on the therapeutic role of **RvD2** in neuropathic pain. It was discovered that **RvD2** alleviated locomotor dysfunction, allodynia, and hyperalgesia in the rat in an in vivo model of spinal cord injury (SCI) and reduced the pro-inflammatory phenotype of microglia, reducing inflammation [46]. A recent report also revealed that following trauma to the sciatic nerves, repeated intrathecal (IT) administration of **RvD2** (500 ng) prevented persistent mechanical allodynia and heat hyper-nociception, and in turn, IT post-treatment with **RvD2**, at the same dose, relieved long-lasting neuropathic pain [47]. What is even more interesting is that repetitive pre-administration of **RvD2** (IT, 500 ng) alleviated the level of inflammatory mediators such as interleukin-17 (IL-17), chemokine ligand 1 (CXCL1) and glial fibrillary acidic protein (GAFP). This indicates the therapeutic role of **RvD2** in spinal inhibition of neuroinflammation. The antidepressive role of **RvD2** was reported in turn in a murine in vivo model of neuropathic pain. The intensity of depression-like behaviour was measured in terms of the duration of immobility (tail suspension test). This test measures the immobility time of animals suspended by their tails. A long immobility time indicates a high level of depression. The immobility time was reduced after intracerebroventricular (ICV) injection of **RvD2** (10 ng) and this antidepressive role of **RvD2** was transduced through activation of mTORC1 signalling [48]. Of interest, the same research group also demonstrated the antidepressant properties of **RvD2** (at the same concentration of 10 ng) in two other mouse models of depression induced using endotoxin from Gram-negative bacteria lipopolysaccharide (LPS) or chronic unpredictable stress [49,50].

### 3.3. Neurodegenerative Diseases

The pro-resolution activity of **RvD2** was also shown in mice with the LPS-induced Parkinson’s disease model. In this animal model, LPS activates microglia to release neurotoxic factors, which then cause progressive dopaminergic neurodegeneration. In these animals, LPS treatment resulted in increased apomorphine-induced behaviour and a reduced level of tyrosine hydroxylase (TH) expression in substantia nigra pars compacta (SNpc). Both unfortunate events were prevented through treatment with **RvD2** (25–100 ng/kg), indicating that **RvD2** protected against the loss of dopaminergic neurons and subsequently improved the behavioural deficit [51]. The impact of **RvD2** on microglial activity was also investigated, which is of great interest because excessively active microglia can be detrimental to neurones. The results presented by Tian et al. supported their previous observations. They showed that when pretreated with **RvD2**, primary microglia cells exhibited reduced expression of proinflammatory cytokines compared to that after LPS [51].

### 3.4. Cardiometabolic and Cardiovascular Diseases

Atherosclerotic lesions are a common chronic inflammation condition characterised by the accumulation of lipids, cholesterol, and fibrous elements in the large arteries, causing an inhibition of blood flow [52]. Resolution signalling in cardiovascular diseases is a clue to treatment success. Spite and Fredman extensively reviewed the RvD2-GPR18 axis in atherosclerotic lesions in 2023 [53]. They analysed earlier studies from many independent research groups, indicating that GPR18 mediates the biological effects of **RvD2**, as shown in gene knockdown, gene knockout mice or synthetic inhibitors. Evidence that the RvD2-GPR18 axis may mediate the regeneration of atherosclerotic lesions was provided by Bardin et al. [54]. This research group showed that the highest expression of GPR18 was observed in the human coronary artery at the early stages of atherosclerosis, which was correlated with a high concentration of **RvD2** compared to healthy coronary arteries. Chronic inflammation that arises when the arteries are affected by atherosclerosis reflects a situation in which the resolution of inflammation is impaired. This could be because the expression of GPR18 is considerably lower in advanced atherosclerosis, which could lead to a decrease in **RvD2** function. In addition, the same research group experimented with ApoE^−/−^ hyperlipidemic mice fed a high-fat diet for 4 weeks to develop an established atherosclerotic lesion burden in the aortic arch and the thoracic aortas. Animals that were intraperitoneally (IP) injected with **RvD2** (100 ng/mouse) three times a week showed a reduced degree of atherosclerosis, while in the presence of **O-1918** (GPR18 antagonist), the positive effects of **RvD2** were not observed [54]. 

Cardiovascular diseases are linked to impairment of blood flow to the heart muscle due to the blockade of the coronary arteries. In this situation, arteriogenesis is a compensatory mechanism that helps to maintain a proper flow. This is a unique process in that existing collateral arterioles are transformed into functional arteries outside the hypoxic area. **RvD2** is endogenously produced during the resolution phase in sterile or infectious inflammation. However, therapeutic administration of **RvD2** could improve the recovery process. In the mouse model of hind limb ischemia (HLI), subcutaneous **RvD2** (100 ng/mouse) significantly improved perfusion recovery by day seven. At the same time, the untreated group showed no improvement by day 14 post-HLI [55]. This positive effect was due to an increase in arteriogenesis. Moreover, **RvD2** modulated the inflammation by reducing the level of neutrophils in the skeletal muscle of mice undergoing HLI, effectively lowering the level of granulocyte-macrophage colony-stimulating factor (GM-CSF) and the proinflammatory cytokine TNF-α, which are induced in ischemia. These effects were connected with improved revascularisation and boosted skeletal muscle regeneration. Further confirmation of the important role played by GPR18 in these processes was provided by demonstrating that GPR18-deficient mice showed no improvement in perfusion recovery during HLI compared with their wild-type littermates. 

Cardiovascular disorders, including hypertension, are associated with vascular damage, endothelium dysfunction, adventitial fibrosis, and inflammation. An interesting methodological solution was presented by the Briones research group, which studied two different groups of mice with hypertension: first, a prevention group in which angiotensin II (AngII)-infused mice were treated with **RvD2** (100 ng/mouse) one day before AngII infusion and throughout the experiment (every two days); second, an intervention group in which **RvD2** was given at day seven after AngII infusion till the end of the experiment [56]. They proved that administration of **RvD2** to hypertensive mice prevented and reduced changes in cardiovascular function, remodelling, fibrosis, immune cell infiltration, and inflammation. Treatment with **RvD2** maintained endothelium-dependent vasorelaxation to acetylcholine in small mesenteric arteries, which was impaired after AngII. **RvD2** prevented the adverse effects of AngII, such as myocardial hypertrophy, fibrosis, and cardiomyocyte apoptosis.

### 3.5. Gastrointestinal Diseases

The role of the endocannabinoid system (ECS) in gastrointestinal disorders is significant and also extremely interesting, as it appears that the increased presence of cannabinoid receptors in certain disorders contributes to protecting the gastrointestinal tract and maintaining its homeostasis [57]. Inflammatory bowel diseases (IBD) such as Crohn’s disease (CD) and ulcerative colitis (UC) are caused by an inappropriate inflammatory response that results in chronic intestinal damage. Colon biopsies from patients with CD and UC and colon tissue from mouse colitis models showed higher levels of GPR18 transcripts compared to healthy controls in the microarray expression data [58]. Although the expression of GPR18 does not affect the course of colitis in mice, a possible ligand interaction with this receptor may play a role in inflammation regulation. In ex vivo culture experiments, it was shown that **RvD2** had a modulatory effect on CD-associated colonic inflammation [59]. Treatment with **RvD2** resulted in a lower level of pro-inflammatory cytokines in the intestinal mucosa explant of the patient with an active form of CD compared to the nontreated biopsies. **RvD2** was as effective as Infliximab, a TNF-α blocker approved by the Food and Drug Administration (FDA), in treating CD or UC. The significance of GPR18 is emphasised in metabolic syndrome, where pro-inflammatory adipocytokines are released by adipose tissue or chronic systemic inflammation, which contributes to the development of cardiovascular disease or diabetes. Chronic inflammation can cause hypothalamic neurons to receive inadequate signals, which may be associated with eating disorders. It has been shown that **RvD2** at the dose of 3 ng/mouse (ICV administration) restored standard organ blood supply and glucose tolerance in obese mice and reduced the release of hypothalamic pro-inflammatory cytokines and calorie intake [60]. 

Irritable bowel syndrome (IBS) is another disorder of the gastrointestinal tract that affects the small intestine, large intestine, and colon. Patients with IBS suffer from diarrhoea and constipation. IBS is accompanied by visceral hypersensitivity related to abnormal perception of visceral stimuli. An important role in the response to these stimuli is played by the Transient Receptor Potential (TRP) channels, among which TRP vanilloid 1 (TRPV1) is the most studied. Inhibition of TRPV1 sensitisation is one way of treating IBS. An in vitro study showed that treated murine dorsal root ganglion (DRG) neurones with higher concentrations of **RvD2** (1 μM) inhibited TRPV1 activation, supporting its analgesic properties. In turn, the lowest tested concentration of **RvD2** (10 nM) prevented histamine-induced TRPV1 sensitisation and reversed existing sensitisation via GPR18 coupled to Gαi proteins [61]. Then, in vivo studies confirmed the observations obtained in vitro in two mouse models of visceral hypersensitivity (VHS), i.e., post-inflammation and post-infectious (mice infected with *C. rodentium*). The two models differ in the visceral motor response (VMR) intensity when post-inflammation VHS showed a stronger VMR to colorectal distension. **RvD2** (10 nM) reduced the VMR to colorectal distension in both models and restored this response to the control level [61]. IBS is not directly related to inflammation as it does not cause visible damage to the digestive tract (unlike inflammatory bowel disease, where the intestinal tract is damaged by inflammation). However, there is growing evidence that inflammation in the gastrointestinal mucosa may play a role in the pathogenesis of IBS [62]. It is suggested that an increase in mast cells may be responsible for this inflammatory response [63]. 

### 3.6. Peritonitis

Peritonitis is a severe medical condition in which the membrane lining the inner abdomen becomes inflamed or infected. If peritonitis is left untreated, the infection can rapidly spread throughout the body, triggering a severe immune response known as sepsis. Recently, it has been reported that **RvD2** could be involved in the action of the inflammasome. The inflammasome is a large intracellular protein complex that activates the pro-inflammatory cascade. Its role in the immune system is to promote cytokine maturation. RvD2 (10 nM) suppressed only the activity of NLRP3, with no effect on AIM2, NALP3, and NLRC4 inflammasomes in activated macrophages [64]. Interestingly, the effect was observed when **RvD2** was administered before and after the inflammasome activation. It indicates that **RvD2** can downregulate the priming and secondary signal induction process during inflammasome activation. In turn, in the mice peritonitis models, **RvD2** (1 μg/mouse) decreased the level of IL-1β, IL-6 and TNF-α in LPS-treated mice but only affected IL-1β in mice injected with monosodium urate or alum [64]. In infectious peritonitis, **RvD2** (100 ng/mouse) given in the late phase of infection (48 h after leakage of faecal bacteria from the punctured caecum into the sterile peritoneum) upregulated the number of the splenic neutrophil and immature populations of granulocytic and monocytic cells indicating that **RvD2** may inhibit potential chronic inflammation [65]. Prior research has shown that intravenously (IV) administered **RvD2** can effectively reduce murine peritonitis at very low dosages, ranging from picograms to nanograms [66].

### 3.7. Periodontitis

Periodontitis is a common human disease called gum disease that affects the soft tissue around the teeth. The body reacts to bacteria that accumulate in the mouth, causing inflammation. If untreated, the inflammation spreads below the gums and along the roots of the teeth and can lead to tooth loss. **RvD2** also has a documented effect in periodontitis in rats that was caused by mechanical exposure of the pulp of molars, which was then left for 3 weeks to develop bacterial infection [67]. An in vivo study showed that intracanal treatment with **RvD2** at a dose of 20 ng resulted in reduced MPO activity. In vitro study showed that **RvD2** (100 nM and 200 nM) improved the mineralisation of root canal apices in dentin primary cells and reduced the periapical lesion size. Evidence that inflammation was reduced was a lower influx of inflammatory cells (polymorphonuclear neutrophils, leucocytes, and monocytes). The molecular mechanisms of action of **RvD2** that heal periapical bone lesions and regenerate pulp-like tissue are based on the enhanced expression of dentin matrix acidic phosphoprotein 1 (DMP1) and the phosphorylation of STAT3. Interestingly, the expression of GPR18 was upregulated in the **RvD2**-treated group of rats with periodontitis. This conclusion is particularly noteworthy because previous research has demonstrated that the interaction between the RvD2-GPR18 on macrophages leads to the phosphorylation of STAT3. This contributes to macrophage phagocytosis, promoting the resolution of inflammation. Of interest is the unique profile of lipid mediators associated with the state of periodontal inflammation. An increase in SPM synthesis activity was noticed in patients with periodontitis before surgical treatment, particularly the increased levels of the D series resolvin pathway markers 4-HDHA, 7-HDHA, and 17-HDHA, as well as their corresponding receptors, in particular GPR18 [68]. The host response may involve an effort to resolve inflammation.

### 3.8. SARS-CoV-2-Induced Inflammation

Coronavirus disease 2019 (COVID-19) is caused by the SARS-CoV-2 virus. Controlling the ‘cytokine storm’ was the biggest challenge during the COVID-19 pandemic. This storm is an uncontrolled and powerful immune system reaction that often causes the death of COVID-19 patients. The FDA authorised the use of Veklury (remdesivir) on 22 October 2020, to treat acute cases of COVID-19 requiring hospitalisation. It was one of the first treatments for severe symptoms of the disease. However, the World Health Organisation (WHO) Guideline Development Group (GDG) of international experts did not recommend using remdesivir [69]. The reason was that the available data did not support the claim that it results in meaningful patient improvements. Remdesivir belongs to a class of drugs called antivirals. These are known to interfere with the natural ability to respond to infection. Analysis of eicosanoid metabolomic changes in rat plasma after remdesivir administration demonstrated that the concentration of **RvD2** and other inflammatory and immunology-related eicosanoids was significantly reduced [70]. An interesting aspect that emerged from the analysis is that naturally occurring **RvD2** weakens pathological thrombosis and helps to remove blood clots. The pathology of COVID-19 leads to complications related to blood vessels, like blocked veins or arteries. These findings support the hypothesis that SPMs play a role in improving the prognosis for recovery. Evidence for this statement is provided by studies in which **RvD2** (10 nM) treatment attenuated the response to virion spike 1 (S1) glycoprotein (an inflammation inducer of SARS-CoV-2) by reducing the release of selected chemokines and cytokines, including interleukin-8 (IL-8) and TNF-α, in macrophages from volunteers with and without cystic fibrosis [71]. The authors also found that **RvD2** restored the expression of miR-29a and miR-125a in S1-treated macrophages, while the activation of transcription factor NF-κB was reduced. It is worth mentioning that in the development and progression of many inflammatory diseases, the stimulation of NF-kB in macrophages is of great importance [72]. Moreover, the studies cited here suggest that **RvD2** may have an unappreciated role in macrophage polarisation. This means that the classical activation of macrophages (M1, pro-inflammatory phenotype) might be inhibited in favour of the alternative activation (M2, anti-inflammatory phenotype). 

### 3.9. Duchenne Muscular Dystrophy

Duchenne muscular dystrophy (DMD) is a genetic disease that affects only boys and is characterised by progressive muscle degeneration. DMD is characterised by the continuous accumulation of inflammatory cells contributing to muscle degeneration through reduced muscle stem cell myogenesis capacity. A properly synchronised inflammatory process has a significant role in restoring normal muscle function after injury. A recent valuable preclinical study demonstrated the therapeutic potential of **RvD2** in different mouse models of DMD [73]. In in vitro approaches, **RvD2** (200 nM) modulates the macrophage phenotype toward anti-inflammatory (M2) phenotype markers CD206, Arginase-1 (Arg1), and CD163), as indicated by the increased expression of genes that are markers of the M2 phenotype, such as *Cd163*, *Pparg*, *Chil3*, and *Anxa1*. These beneficial effects of **RvD2** were confirmed in DMD (mdx mice) mouse models. When **RvD2** (5 μg/kg) was injected systemically into dystrophic mice for 7 and 21 days, it was observed that the percentage of anti-inflammatory macrophages (F4/80+/CD206+) was higher in **RvD2**-treated mice compared to the control group. The results were similar to those of the gold standard treatment for DMD—prednisone. Anti-inflammatory cytokines produced by M2 cells promote myogenesis and angiogenesis and induce the synthesis of extracellular matrix (ECM) components, which are critical for properly and efficiently forming new muscle fibres [74]. This fact was confirmed in the cited study since **RvD2** stimulated skeletal muscle regeneration by increasing the number of Myog+ differentiated myoblasts. It is worth noting that a critical overtone of this research is that these effects are GPR18-dependent. When the receptor was knocked out, knocked down, or blocked by the pharmacological antagonist **O-1918**, the positive therapeutic effect of **RvD2** was not observed. 

### 3.10. Asthma and Lung Inflammation

Asthma is a chronic inflammatory lung disease that leads to excessive contraction of the bronchi, which in turn causes a feeling of breathlessness, difficulty breathing, or tightness in the chest. In a mouse experimental model of asthma (house dust mite (HDM) challenge), **RvD2** (100 ng) decreased the number of eosinophils, infiltrating macrophages, exudative macrophages, and lymphocytes in bronchoalveolar lavage, thereby accelerating the natural resolution of inflammation, which took more than 3 days in untreated mice [75]. To confirm that **RvD2**-induced effects are results of GPR18 modulation, the antagonist **O-1918** was administered. This led to an increase in CD4 T-lymphocytes, IL-5, and IL-13 (in vivo) and Th2-type immune response cytokines and total cell counts in bronchoalveolar lavage (ex vivo). The results confirm that the resolution of inflammation is mediated by signalling in the RvD2-GPR18 axis. The authors also reported higher expression of GPR18 on peripheral blood eosinophils of patients with severe asthma. This might indicate an important role played by GPR18 in regulating the immune response in this pathological condition. Moreover, histopathological analysis revealed that the damages in bronchial epithelium, airway epithelial mucous, and peribronchial and perivascular leukocyte infiltration associated with inflammation were diminished. The hallmark of an ongoing inflammatory process in the lungs is the adhesion of leukocytes to vascular endothelium [76]. A properly functioning vascular endothelium prevents leukocyte and neutrophil adhesion and migration. It also prevents excessive permeability of the vessel walls. In the presence of pathological factors, this functional balance of the endothelium is disturbed, leading to endothelial dysfunction. It has been shown that neutrophil-like HL60 cells in the presence of **RvD2** (50 ng/mL) adhered less firmly to the inflamed HUVEC cells layer and transmigrated across the blood vessel endothelial barrier [77]. **RvD2** produced an identical effect in LPS-challenged mice where adhesion proteins on neutrophils were downregulated, and neutrophil apoptosis was upregulated, positively affecting the inflammation process [77]. Neutrophils have multidirectional effects on the host defence response. They can have a positive effect by entering the apoptosis pathway, which creates conditions for macrophage stimulation towards an anti-inflammatory profile. However, neutrophils can be responsible for tissue damage when they overproduce reactive oxygen species or proteases. This so-called neutrophilic inflammation can be regulated by efferocytosis, the phagocytosis of neutrophils by macrophages. This process is an indispensable part of the proper resolution of inflammation. Mice with acute lung inflammation, when injected with **RvD2** (100 ng/mouse), exhibited enhanced phagocytosis of apoptotic neutrophils by macrophages [77]. The mechanism of action of **RvD2** described here may be precious in diseases such as autoimmune diseases or atherosclerosis, where inefficient phagocytosis is at the root of chronic inflammation.

### 3.11. Placenta Disorders

It is noteworthy that, from the studies to date, it can be concluded that **RvD2** has a considerable influence on blood vessel and muscle tissue function. The combination of these two areas is reflected in the dysfunction of the human placenta. The expression of GPR18 was confirmed in vascular smooth muscle (VSM) and extravillous trophoblast (EVT), which may indicate the role of SPMs in pregnancy, perinatal inflammation, and vascular injury [78]. These results are consistent with those of Ulu et al., who confirmed GPR18 expression at both transcript and protein levels in human placental samples, which were collected following delivery from mothers in their third trimester at the time of delivery [79]. In this study, **RvD2** (1 and 100 nM) decreased the level of proinflammatory IL-6 in human umbilical artery smooth muscle cells (HUASMC) stimulated with LPS. In contrast, the level of GPR18 expression in this condition was significantly upregulated. The action of **RvD2** was also assessed in an in vitro study of normal human immortalised placental trophoblasts (HTR-8) stimulated with TNF-α. There was a slight decline in IL-1β and IL-6 levels in placental trophoblasts in the presence of TNF-α, but the change was not significant [79]. Because placental inflammation contributes to abnormal embryonic heart development [80], preterm delivery or pregnancy loss [81], the outcomes above could indicate a crucial role for **RvD2** on placental function and, therefore, offspring health. However, these studies have several limitations, such as the fact that the stimulation with inflammatory factors was performed in cell lines and not in placental explants. The limitation of the methodology used by Ulu et al. is the lack of data on placentas of different trimesters. 

A summary of the biological activity of RvD2 is presented in Table 1.

## 4. Non-SPM Modulators

Another molecule of great interest is **NAGly**, even though it cannot be categorised as an SPM. This endogenous metabolite of endocannabinoid anandamide was described as an efficacious agonist in GPR18 [25]. However, signal transduction of NAGly appears to be dependent on the cell type or assay system used. In glioblastoma multiforme cell lines with endogenous GPR18, **NAGly** did not elicit phosphorylation (pERK1/2). Interestingly, proof of the GPR18–NAGly interaction was found in a β-arrestin experiment using CHO cells transfected with GPR18 but was not present when expressed in human embryonic kidney 293 cells (HEK293) [25]. Based on these reports, it is worth looking at NAGly’s action in the context of anti-inflammatory activity. Interesting results were presented by Takenouchi et al. [82]. It was shown that in highly GPR18 mRNA-expressing macrophages (pro-inflammatory phenotype M1), **NAGly** strongly induced apoptosis. Instead, no effect was observed in macrophages with anti-inflammatory phenotype M2, which exhibited lower GPR18 expression. These results only highlight the relevant role played by GPR18 in resolving inflammation indirectly by stimulating macrophages, which can reduce inflammation via tissue repair and fibrosis. Another study reported that **NAGly** plays a role in modulating inflammation in the brain through various mechanisms. Of interest was the experiment in which the level of prostaglandin J2 (PGJ2) and lipoxin A_4_ (LXA_4_)—two molecules which play a pivotal role in the resolution phase of inflammation—were upregulated by **NAGly** in HEK293 GPR18-transfected cells. As a result of **NAGly** action, there was also an increase in cell death, which targets pro-inflammatory cells to resolve the inflammatory response [14]. 

The role of endothelial cells (ECs) in inflammation is also worth highlighting. Endocannabinoid metabolic enzymes make ECs a unique target for inflammatory disorder therapies and the expression of GPR18, GPR55, and CB1 receptors [83]. The vascular endothelium is crucial in maintaining the body’s homeostasis as it produces compounds that affect blood coagulation and fibrinolysis, as well as substances involved in regulating inflammatory processes. Damage to the vascular endothelium can lead to the development of civilised diseases, such as atherosclerosis and hypertension. Synthetic agonists of GPR18 **PSB-MZ1415** (also known as **PSB-KK-1415**) and **PSB-MZ1440** (also known as **PSB-KK-1440**) promoted endothelium-dependent relaxation of human pulmonary arteries that express GPR18, and these effects were partially blocked by synthetic GPR18 antagonist **PSB-CB27** [84]. In turn, Fabisiak et al. revealed that **PSB-KK-1415** reduced the expression of TNF-α in a semi-chronic mouse model of colitis induced by 2,4,6-trinitrobenzene sulfonic acid (TNBS), and the tissue of distal colon was characterised by less damage when compared to the control group [85]. Interestingly, the level of MPO was reduced in the inflamed mouse colon by both agonists and antagonists of GPR18. While the agonist activity is evident and confirmed in other reports, the antagonist activity is unclear and subject to further investigation. Nevertheless, the anti-inflammatory activity of the GPR18 agonist has once again been confirmed. 

Another synthetic non-lipid agonist of GPR18 was shown to be beneficial in reducing inflammatory markers. According to research conducted by Dort et al., both in vitro and in vivo investigations found that **PSB-KD107** had a beneficial effect in a DMD model [86]. First, the authors showed that GPR18 is present in human myogenic cells isolated from the blood of DMD patients. The results demonstrated that the application of **RvD2** (200 nM) had a comparable effect to that of **PSB-KD107** (10 μM) on enhancing the myogenic capabilities of these cells. In vivo experiments were conducted for 21 days in mdx mice, during which **PSB-KD107** (1 mg/kg) and **RvD2** (5 μg/kg) were injected once a week. The analysis of muscle strength in a live setting, following a 3-week treatment, demonstrated the effectiveness of **PSB-KD107**, which was comparable to that of **RvD2**. Next, this study used a GPR55 agonist, compound **O-1602** (5 mg/kg), to test whether stimulation of this receptor could also benefit DMD. Results showed no considerable effect on muscle strength. **PSB-KD107** has also been reported to have a vasorelaxant effect and antioxidant activity [87]. This outcome is of great interest as abnormalities in both vasodilation and vascular function are a common problem in chronic diseases such as rheumatoid arthritis, lupus, and IBS. A summary of the biological activity of the small molecule ligands of GPR18 is presented in Table 2.

## 5. Materials and Methods

The following literature databases were searched up to 15 January 2024: PubMed, Scopus, and Web of Science. We used the entry words “GPR18”, “inflammation”, “resolution”, and “resolvin D2”. We considered only the most recent sources relevant for citing (no more than 5 years old). This applies to the biological activity of RvD2 and other ligands.

## 6. Conclusions

**Figure 7:**
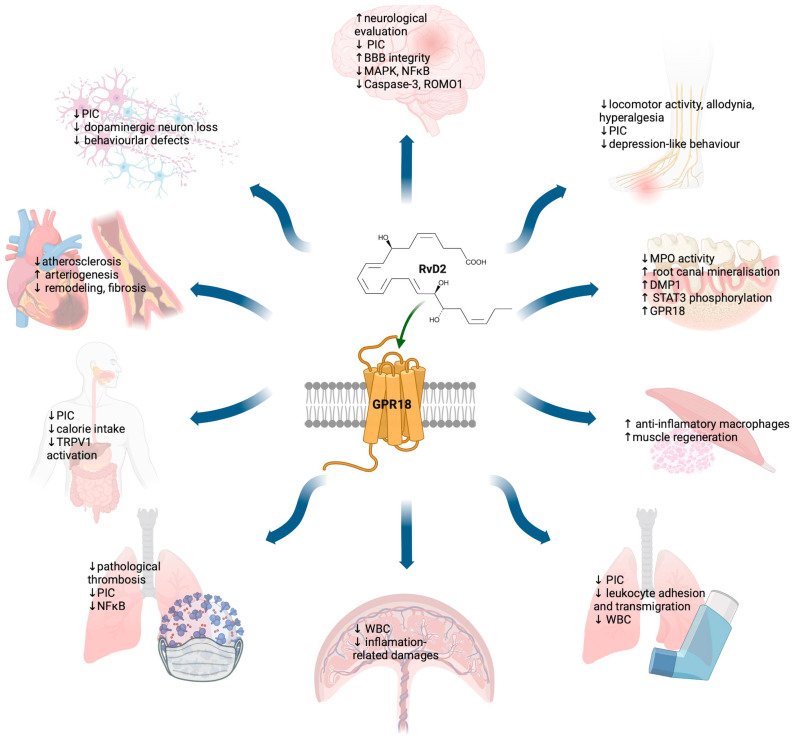
Omnidirectional effect of GPR18 activation in the resolution of inflammatory responses. PIC—pro-inflammatory cytokines; BBB—blood-brain barrier; MAPK—mitogen-activated protein kinase; NF-κB—nuclear factor kappa B; ROMO1—reactive oxygen species modulator 1; TRPV1—transient potential receptor vanilloid 1; DMP1—dentin matrix acidic phosphoprotein 1; WBC—white blood cells; MPO—myeloperoxidase. ↑ stimulatory/increased effect; ↓ downregulated/inhibited effect. Created with BioRender.com, accessed on 27 January 2024.

This review highlights the biological intervention of **RvD2** and its therapeutic potential to modulate GPR18. The role of the GPR18-RvD2 axis is discussed in relation to ongoing inflammation and the resolution of inflammation. The action of **RvD2** in these conditions has positive effects on wound healing, cardiometabolic diseases (cardiovascular disease and diabetes), mild cognitive decline and mood disorders, pulmonary diseases (allergy, asthma), and arthritis (Figure 7). 

However, there are many challenges associated with this type of therapeutic intervention. **RvD2** is a highly potent lipid mediator categorised as immunoresolvent. The body produces it as part of a natural response to acute inflammation. 

Although numerous data proved that, when given exogenously to animals suffering from a particular health condition connected with chronic inflammation, it partially supports the host’s defence through its ability to enhance the phagocytic capacity of macrophages, e.g., decreasing pro-inflammatory cytokines (PIC). That is because converting dietary omega-3 and omega-6 fatty acids is multi-step and complex and sometimes too slow and inefficient to resolve inflammation in a reasonable time. Moreover, its polyunsaturated structure makes this molecule chemically and biologically unstable. Numerous data cited in this review do not seem to support this hypothesis. It is worth noting that in in vivo studies, when **RvD2** is exogenously administered, no attention is paid to the level of its metabolite. It is unclear whether this positive effect on inflammation is due to **RvD2** or its breakdown products. **RvD2** is a polyunsaturated, unstable compound with three Z- and three E-configured isomers with three chiral centres. **RvD2** must be protected from temperature, moisture, and air, and stored at −80 °C according to the supplier’s instructions. Studies have demonstrated that RvD2 can undergo chemical modifications that affect its activity, including changes in isomer configuration, enantiomerisation, and oxidation [66]. These modifications can occur in the biological environment due to biological mediators, whether in vitro or in vivo. One solution to this problem could be the use of small molecule ligands. However, Ye et al. [90] described new insights into cannabinoid receptor signalling that should be considered. These include the complex binding pattern to GPR18, which can occur orthosterically (lipid-like) or allosterically, and the chemical nature of ligands, which can be agonists or antagonists or partially active agonists and antagonists. Biased activity of ligands should also be considered, as ligands can stabilise different receptor configurations and lead to different interactions with intracellular signal proteins. Another issue is that the results obtained by different research groups are now inconsistent and sometimes controversial, which might be due to the lack of selective pharmacological tools (GPR18 is still an orphan receptor), the use of different cellular models (including GPR18-overexpressing cell lines), and biased downstream effects, impacting cellular responses. There is still a lot of work that needs to be completed. Firstly, it should be carefully verified whether the effects induced by **RvD2** are indeed the results of GPR18 stimulation. If this is the case, the effect should be blocked in the presence of receptor antagonists. Finally, it is necessary to check if the activity of **RvD2** can be replaced by more stable small molecule ligands. 

## Figures and Tables

**Table 1:** Summary of the selected biological activity of RvD2.

Target Diseases	Type of Study	Type of Cells/Host	Dose	Effects/Mechanism of Action	Ref.
subarachnoid haemorrhage (SAH)	in vivo	male Sprague–Dawley rats (with endovascular perforation)	0.9 μg/kg single, IN ^a^ administration	▪ improved motor and sensory function ▪ inhibited mast cell degranulation ▪ downregulated MMP-9, AQP4, caspase-3, ROMO1 ▪ decreased APP ▪ increased MBP	[41]
diabetes mellitus (DM)-related acute ischemic stroke (AIS)	in vivo	male C57BL/6J mice (high fat diet+streptozotocin)	1 nM	▪ mitigated brain injury, neurological dysfunction, the inflammatory response ▪ increased CD206/iNOS ratio ▪ downregulated MAPK and NF-κB	[42]
	in vitro	macrophages from AIS patient			
cerebral ischemia/reperfusion (CI/R) injury	in vivo	male Sprague-Dawley rats (after middle cerebral artery occlusion)	50–100 μg/kg IP ^b^ single administration	▪ decreased IL-6, TNF-α ^e^	[44]
spinal cord injury (SCI), neuropathic pain	in vivo	male Sprague-Dawley rats (after laminectomy)	50 ng/kg IT ^c^ administration for 7 days	▪ alleviated locomotor dysfunction, allodynia, hyperalgesia	[46]
chronic constriction injury (CCI), neuropathic pain	in vivo	male C57BL/6J mice	500 ng/mouse IT administration on days 4, 5 and 6 following the CCI or single administration on day 14 following the CCI	▪ prevented persistent mechanical allodynia, heat hyper-nociception ▪ relieved long-lasting neuropathic pain ▪ alleviated the level of IL-17, CXCL1, GAFP	[47]
neuropathic pain	in vivo	male BALB/c mice (after unilateral spared nerve injury)	10 ng ICV ^d^ administration	▪ reduced immobility time in the tail suspension test	[48]
Parkinson’s disease	in vitro	primary microglia,	1.25–20 μM	▪ reduced level of IL-18, IL-6, NO, TNF-a and IL-1b ▪ inhibited TLR4/MyD88 signal pathway ▪ reduced ROS	[51]
	in vivo	male Sprague-Dawley rats (LPS-injected)	25–100 ng/kg single injection	▪ decreased apo-morphine-induced rotational cycles ▪ retained TH-positive neurons in SNpc	
atherosclerotic lesions	in vivo	ApoE+/+ C57BL/6J mice and ApoE^−^/^−^ C57BL/6J mice	100 ng/mouse IP administration 3 times/week for 4 weeks	▪ decreased atherosclerotic lesion size and necrotic core areas ^e^	[54]
hind limb ischemia (HLI)	in vivo	male C57BL/6J mice (after transection of the femoral artery and vein)	100 ng/mouse subcutaneously administration daily for 14 days	▪ improved perfusion recovery by day 7 ▪ reduced level of neutrophils in skeletal muscle ▪ decreased GM-CSF, TNF-α ▪ improved endothelial cell migration ^e^	[55]
hypertension	in vivo	C57BL/6J mice (infused with angiotensin II)	100 ng/mouse every second day for 14 days	▪ prevented myocardial hypertrophy, fibrosis, cardiomyocyte apoptosis, immune cell infiltration and inflammation ▪ increased expression of M2 anti-inflammatory markers *Hmox-1* and *Cd163*	[56]
Crohn’s disease (CD)	ex vivo	colonic biopsy	not given	▪ decreased IL-β	[59]
obesity	in vivo	male Swiss mice (on a high-fat diet)	3 ng/mouse or 50 ng/mouse ICV administration daily for 11 days	▪ reduced body mass gain (3 ng) ▪ reduced visceral fat (3 ng and 50 ng) ▪ improved glucose tolerance (3 ng) ▪ increased IL-6 and IL-10 (3 ng)	[60]
irritable bowel syndrome (IBS)	in vitro	dorsal root ganglion (DRG) neurons	10 nM or 1 μM	▪ inhibited TRPV1 activation (1 μM) ▪ prevented histamine-induced TRPV1 sensitisation (10 nM) ▪ reduced visceral motor response to colorectal distension	[61]
	in vivo	BALB/c mice with post-infectious or post-inflammation visceral hypersensitivity	300 ng/mouse IP administration every other day for 1 week		
periodontitis	in vivo	male Wistar rats	20 ng/mouse administration directly to the root canal	▪ reduced MPO activity ▪ reduced the periapical lesion size ▪ calcified root canal apices ▪ induced root apex closure ▪ reduced inflammatory cell accumulation in periapical tissues	[67]
COVID-19	in vitro	macrophages from peripheral blood	10 nM	▪ reduced IL-8, TNF-α and NF-κB ▪ restored the expression of miR-29a and miR-125a	[71]
Duchenne muscular dystrophy (DMD)	in vivo ex vivo in vitro	male mdx mice muscles single myofibers, monocytes/macrophages	5 μg/kg/day for 7 or 21 days IP administration 200 nM	▪ increased level of anti-inflammatory macrophages (F4/80+/CD206+) ▪ increased contractile properties ▪ increased myogenesis ^e^	[73]
asthma	in vivo	Balb/c mice (intratracheal administration of house dust mite extract)	100 ng/mouse IN administration on days 15 and 16 after sensitisation with house dust mites	▪ decreased number of eosinophils, infiltrating macrophages, exudative macrophages and lymphocytes ^e^ ▪ decreased T2 cytokines (IL-4, IL-5, IL-13) ^e^ ▪ decreased lung expression of E-selectin, ICAM-1, IL-33, Ccl5 RANTES, IL-13, periostin	[75]
lung inflammation	in vitro in vivo	co-culture of HL60 and HUVEC cells CD1 mice	50 ng/mL 100 ng/mouse IV administration	▪ inhibited leukocyte adhesion and transmigration ▪ downregulated adhesion proteins on neutrophils ▪ increased neutrophil apoptosis ▪ improved alveolar morphologies ▪ prevented the production of inflammatory cytokines in circulation and the lungs	[77]
placenta disorder	in vitro	HUASMC HTR8	1 nM or 100 nM 100 nM	▪ decreased IL-6 in the presence of LPS ▪ decreased IL-1β and IL-6 in the presence of TNF-α	[79]
peritonitis	in vivo	mice injected with LPS, monosodium, or alum	1 μg/mouse single IP administration	▪ decreased IL-1β, IL-6 and TNF-α in LPS-treated mice, but only affected IL-1β in mice injected with monosodium or alum	[64]
	in vitro	macrophages with induced inflammasome	10 nM	▪ suppressed NLRP3 inflammasomes (no effect on AIM2, NALP3, NLRC4 inflammasomes)	
infectious peritonitis and secondary lung infection	in vivo	male C57BL/6 mice (after cecal ligation and puncture)	100 ng/mouse via tail vein	▪ increased splenic neutrophil and immature populations of granulocytic and monocytic cells ▪ no effect on pro-inflammatory cytokines	[65]

^a^ IN^—^intranasal; ^b^ IP—intraperitoneal; ^c^ IT—intrathecal; ^d^ ICV—intracerebroventricular; ^e^ the effects blocked by GPR18 antagonist O-1918.

**Table 2:** Summary of the selected biological activity of small molecules ligands of GPR18.

Compound	Type of Study	Test	Dose/s	Observed Effect	Ref.
O-1602	in vivo	diet-induced obesity (Sprague–Dawley rats)	5 mg/kg for 6 weeks IP ^a^	▪ reduced body weight and fat ▪ improves albuminuria ▪ adverse side effects on liver	[88]
	in vivo	diet-induced obesity (Sprague–Dawley rats)	5 mg/kg for 6 weeks IP	▪ no changes in markers of oxidative capacity or adiponectin signalling	[89]
PSB-KD107	in vitro	influence on rat aorta precontracted with phenylephrine (Wistar rats)	----	▪ vasorelaxant effects on endothelium-intact rat aortic rings pIC_50_ = 5.22	[87]
	in vitro	platelet aggregation test (Wistar rats)	0.1 mM	▪ no effect	
	in vitro	ferric reducing antioxidant power (FRAP) assay	0.1–1 mM	▪ antioxidant activity ▪ every 0.1 mM reduced 131 µM of Fe^3+^ ▪ 60–80% activity of ascorbic acid	
	in vitro	2,2-Diphenyl-1-picryl-hydrazyl-hydrate free radical (DPPH) assay	0.1 mM 1 mM	▪ lack of antioxidant activity	
	in vivo	influence on blood pressure (normotensive Wistar rats)	single (10 mg/kg, IP) multiple (8 days, once daily 10 mg/kg, IP)	▪ decreased blood pressure ▪ lack of significant effect	
	in vivo	the effect on normal electrocardiogram (Wistar rats)	single (10 mg/kg IP) multiple—8 days, once daily 10 mg/kg IP	▪ lack of significant effect ▪ lack of significant effect	
	in vivo	inflammation in dystrophic mdx mice (C57BL/10)	1 mg/kg for 3 weeks, IP	▪ reduction of inflammation ▪ improvement in muscle functions ▪ enhancement of myogenesis	[86]
PSB-MZ-1415	in vitro	isolated human pulmonary arteries (hPAs)	---	▪ full relaxation of hPAs precontracted with U46619 (the thromboxane A_2_ analogue) ^c^ ▪ pEC_50_ = 5.2	[84]
	in vivo	forced swim test (Albino Swiss mice)	30 mg/kg, IP	▪ significant decrease in immobility time	[32]
	in vivo	four-plate test (Albino Swiss mice)	1 mg/kg, IP	▪ significant increase in the number of spontaneous punished crossings	
	in vivo	hot plate test (Albino Swiss mice)	1 mg/kg, 3 mg/kg, 10 mg/kg, 30 mg/kg, IP	▪ lack of analgesic properties	
	in vivo	oxaliplatin-induced neuropathic pain (oxaliplatin 10 mg/kg, IP) (Albino Swiss mice) von Frey test 3 h: early-phase allodynia 7 days: late-phase allodynia cold plate test	30 mg/kg, IP	▪ reduction of late-phase tactile allodynia ▪ lack of influence on cold hyperalgesia	
	in vivo	food intake test (Albino Swiss mice)	30 mg/kg, IP	▪ significant reduction of food intake during 2 h	
	in vivo	semi-chronic TNBS-induced colitis (balb/C mice)	1 mg/kg for 4 days (once or twice daily), ^b^ IC	▪ reduction of macroscopic score ▪ expression of TNF-α	[85]
	in vivo	chronic TNBS-induced colitis (balb/C mice)	1 mg/kg for 7 days (once daily), IC	▪ decreased macroscopic score ▪ decreased myeloperoxidase activity	
	in vivo	mustard oil-induced pain (balb/C mice)	1 mg/kg for 4 days, IC	▪ decreased pain-induced behaviours	
PSB-MZ-1440	in vitro	isolated human pulmonary arteries	---	▪ full relaxation of hPAs precontracted with U46619 (the thromboxane A_2_ analogue) ^c^ ▪ pEC_50_ = 4.9	[84]

^a^ IP—intraperitoneal; ^b^ IC—intracolonic; ^c^ the effects blocked by GPR18 antagonist **PSB-CB27**.

## Data Availability

No new data were created or analysed in this study. Data sharing is not applicable to this article.

## Abbreviations

**Abbreviation**	**Meaning**
17HDHA	(±)17-hydroxy-4Z,7Z,10Z,13Z,15E,19Z-docosahexaenoic acid
4HDA	(±)4-hydroxy-5E,7Z,10Z,13Z,16Z,19Z-docosahexaenoic acid
7HDHA	(±)7-hydroxy-4Z,8E,10Z,13Z,16Z,19Z-docosahexaenoic acid
AIS	acute ischemic stroke
AngII	angiotensin II
APP	beta-amyloid precursor protein
AQP4	aquaporin-4
Arg1	arginase-1
BBB	blood-brain barrier
CD	Crohn’s disease
CHO	Chinese hamster ovary cells
CI/R	cerebral ischemia/reperfusion
CNS	central nervous system
COVID-19	coronavirus diseases 2019
CXCL1	chemokine ligand 1
DAMPs	damage-associated molecular patterns
DM	diabetes mellitus
DMD	Duchenne muscular dystrophy
DMP1	dentin matrix acidic phosphoprotein 1
DRG	dorsal root ganglion
ECM	extracellular matrix
ECS	endocannabinoid system
ECs	endothelial cells
EVT	extravillous trophoblast
FAAH	fatty acid amide hydrolase
FDA	food and drug administration
FDA	Food and Drug Administration
GAFP	glial fibrillary acidic protein
GDG	Guideline Development Group
GM-CSF	granulocyte macrophage colony stimulating factor
GPR18	G-protein coupled receptor 18
HEK293	human embryonic kidney 293 cells
HLI	hind limb ischemia
HTR-8	normal human immortalised placental trophoblasts
HUASMC	human umbilical artery smooth muscle cells
IBD	inflammatory bowel diseases
IBS	irritable bowel syndrome
ICV	intracerebroventricular
IL-1β	interleukin-1β
IL-6	interleukin-6
IL-8	interleukin-8
IN	intranasal
IT	intrathecal
IV	intravenously
LPS	*lipopolysaccharide*
LTB_4_	leukotriene B_4_
LXA_4_	lipoxin A_4_
M1	pro-inflammatory phenotype
M2	anti-inflammatory phenotype
MAPK	mitogen-activated protein kinase
MBP	myelin basic protein
MCAO/R	middle cerebral artery occlusion and reperfusion
MMP-9	metalloproteinase-9
MPO	myeloperoxidase
NAGly	N-arachidonoylglycine
NF-κB	nuclear factor kappa B
PGJ2	prostaglandin J2
ROMO1	reactive oxygen species modulator 1
RvD2	resolvin D2 (7S,16R,17S-trihydroxy-4Z,8E,10Z,12E,14E,19Z-docosahexaenoic acid)
SAH	subarachnoid hemorrhage
SCI	spinal cord injury
SNpc	substantia nigra pars compacta
SPMs	specialised pro-resolving mediators
TH	tyrosine hydroxylase
TNBS	2,4,6-trinitrobenzenesulfonic acid
TNF-α	tumor necrosis factor alpha
TRP	Transient Receptor Potential
TRPV1	transient potential receptor vanilloid 1
UC	ulcerative colitis
VHS	visceral hypersensitivity
VMR	visceralmotor response
VSM	vascular smooth muscle
WHO	World Health Organisation
